# A Systematic Review on the Toxicology of European Union-Approved Triazole Fungicides in Cell Lines and Mammalian Models

**DOI:** 10.3390/jox15060208

**Published:** 2025-12-05

**Authors:** Constantina-Bianca Vulpe, Adina-Daniela Iachimov-Datcu, Andrijana Pujicic, Bianca-Vanesa Agachi

**Affiliations:** 1 Department of Scientific Research in Biology, Advanced Environmental Research Institute, West University of Timisoara, Oituz 4, 300086 Timisoara, Romania; constantina.vulpe@e-uvt.ro; 2 The Academy of Romanian Scientists, Ilfov 3, 050044 Bucharest, Romania; andrijana.pujicic@e-uvt.ro (A.P.); bianca.agachi@e-uvt.ro (B.-V.A.); 3 Department of Biology, Faculty of Chemistry, Biology, Geography, West University of Timisoara, Pestalozzi 16, 300115 Timisoara, Romania

**Keywords:** fungicidal triazole class pesticides, human health toxicity, in vitro cytotoxicity, in vivo mammal studies, molecular mechanisms, ADME, network analysis

## Abstract

Triazole fungicides are widely used in agriculture but may pose risks to human health through occupational, accidental, or environmental exposure. This systematic review aimed to evaluate the toxicity of ten European Union-approved triazole fungicides in rodent models and cell lines. A total of 70 studies were included, reporting quantitative in vivo oral, dermal, or inhalation toxicity in mammals or quantitative in vitro cytotoxicity in human or mammalian cell lines; the exclusion criteria comprised publications not in English or not accessible. Literature searches were conducted in Web of Science, Google Scholar, and the Pesticide Properties DataBase (PPDB), and risk of bias in included studies was assessed using ToxRTool. Due to heterogeneity in study designs, reporting formats, and endpoints, data were synthesized descriptively. Quantitative endpoints included LD_50_/LC_50_ values for in vivo studies and LOEC, IC_50_, LC_50_, and EC_50_ values for in vitro studies, while mechanistic endpoints highlighted apoptosis, oxidative stress, genotoxicity, and endoplasmic reticulum stress. Difenoconazole and tebuconazole were the most extensively studied compounds, whereas several triazoles had limited data. The limitations included heterogeneity of data and incomplete reporting, which restrict cross-study comparisons. Overall, the findings provide a comprehensive overview of potential human health hazards associated with EU-approved triazole fungicides and highlight critical knowledge gaps. The review was registered in Open Science Framework.

## 1. Introduction

Pesticides are nowadays considered essential compounds in sustainable agriculture, ensuring food security by supporting both the quality and quantity of agricultural yields. They comprise a broad range of substances with specific properties designed to protect crops from various pests, including unwanted plants (herbicides), animals (insecticides, rodenticides, and acaricides), and fungi (fungicides). Nevertheless, their extensive use also leads to continuous exposure of both the environment and humans to these compounds. Due to their diverse chemical structures and mechanisms of action, pesticides may exert harmful effects on human health and the environment, thereby impacting the holistic One Health concept [1,2].

Chemical food safety is a critical principle for public health at the global level. Various organizations, such as the World Health Organization, have developed principles and methods for assessing the risks posed by pesticides [3]. Furthermore, the status of chemical contamination in food—such as pesticide residues—is monitored periodically to maintain control over exposure [4].

At the European level, the European Union (EU) has developed one of the most detailed and comprehensive frameworks for ensuring chemical safety in food [5]. In this framework, pesticide use is regulated by EU Pesticide Regulation (EC) No. 1107/2009 [6] concerning the placing of plant protection products on the market, as well as EU Directive 2009/128/EC [7], regarding community action to achieve the sustainable use of pesticides. Other plans encompass regulations for organic farming and the adoption of integrated pest management strategies [8,9]. Maximum residue limits for pesticides in food and feed are established under Regulation (EC) No. 396/2005 [10], providing legally defined thresholds to ensure food safety. Furthermore, the European Union publishes an annual report on pesticide residues in food [11].

Fungicides are a class of pesticides widely used to protect agriculturally important plants from fungal diseases that can compromise both crop yield and product quality. Triazole fungicides can be classified as a group of compounds that contain a triazole ring in their chemical structures. Their mechanism of action is based on the inhibition of lanosterol 14α-demethylase, an essential enzyme in the ergosterol-biosynthesis pathway. By blocking this step, triazole fungicides disrupt fungal cell-membrane formation and impair growth. Evidence from in silico and in vitro studies shows strong binding of triazole derivatives to this enzyme, marked reductions in ergosterol content, and ERG11 gene downregulation in *Candida albicans*, confirming their sterol-biosynthesis-inhibitory activity [12,13,14,15].

Triazole fungicides are frequently utilized in agriculture to protect a broad range of economically important crops from fungal diseases. However, their extensive application also raises concerns regarding potential environmental contamination and human exposure. Considering that we are in the One Health Era, a thorough analysis of both environmental and human health effects is essential. Since triazole fungicides can have diverse impacts on the environment and human health, this study focuses on their potential effects on humans as part of the One Health framework. It is important to note that not all triazole fungicides are approved for use within the European Union. Therefore, this study focuses specifically on the triazole fungicides authorized for use in the EU.

There is an extensive body of review literature addressing various aspects of pesticides, which include topics such as their utility and importance in agriculture and human society [16,17], environmental and food contamination [18,19,20,21,22], analytical methods for their detection in environmental and food matrices [23,24,25], methods of remediation [26,27], human exposure [1,28,29,30], toxicological effects on both the environment [22,31,32,33,34,35] and human health (including mammalian models) [30,33,34,35,36,37,38], as well as regulatory perspectives [2,39]. However, although some of these reviews briefly mention triazole fungicides or provide examples from this class [2,16,17,20,21,25,26,27,30,33,39], only a few include a detailed analysis of fungicides [22,29,31,32,34,35], and, to the best of our knowledge, openly accessible papers with a detailed analysis specifically of triazole fungicides appear to be limited.

In terms of the literature on fungicides, the number of review studies is smaller than that for pesticides in general. These works cover various aspects of fungicides, including their mechanisms of action [40,41], effects on target human fungal pathogens [42], development of resistance [40], metabolism and environmental fate [43], methods of removal or degradation [43], environmental contamination and ecotoxicology [40,41,43,44,45,46,47], and impacts on human health [48,49]. Among these, only a limited number include or address triazole fungicides in detail [40,42,44], rather than focusing solely on a few examples from this class, while some reviews may not address them at all.

Review studies focusing primarily on triazole fungicides are limited in number. The few existing studies examine aspects such as their toxicity to target fungal species [50,51], environmental toxicity [12,52,53,54], and effects on human health and laboratory mammal models [55,56,57,58].

The limited number of review studies specifically addressing triazole fungicides is also reflected in the results obtained from the Web of Science database (as of 12 November 2025) [59]. Using the Advanced Search and Query Builder functions with the key search phrases (TI = (pesticide)) AND TI = (review), (TI = (fungicide)) AND TI = (review), and (TI = (triazole fungicide)) AND TI = (review), it was determined that articles containing the terms fungicide and review in their title represent only 3.6% of those containing pesticide and review. Moreover, articles including triazole fungicide and review account for merely 8.4% of those with fungicide and review, and only 0.3% of those with pesticide and review. In total, only six such articles were identified, of which only one report effects on human health or mammalian models [55].

Given the limited number of existing review studies specifically addressing triazole fungicides and the methodological constraints observed in those available—including the restricted number of compounds analyzed, the lack of quantitative and comparative in vivo–in vitro assessments, and the predominantly narrative approach without adherence to systematic frameworks such as PRISMA—the present review is both timely and necessary. It aims to systematically integrate and critically evaluate current data on triazole fungicides, encompassing different exposure types, toxicological endpoints, and experimental models. This comprehensive synthesis seeks to provide a more robust understanding of their potential effects on human health and the environment, contributing to the broader One Health perspective and supporting evidence-based regulatory and scientific advancements.

The scope of this study is to systematically evaluate the human health toxicity of European Union-approved triazole fungicides. This review integrates evidence from in vitro studies in mammalian cell lines and in vivo studies in rodent models, encompassing oral, dermal, and inhalation exposure routes. Both quantitative data, such as dose–response relationship values, and qualitative data, including mechanistic insights into toxicity pathways, are systematically evaluated in this review. This study stands out by providing a thorough and comparative assessment of the toxicological effects of EU-approved triazole fungicides, addressing a significant knowledge gap given that limited studies have comparatively assessed these compounds, and even fewer focus specifically on human health. The objectives of this study comprise:

1. Identification and characterization of the EU-approved triazole fungicides;
2. Identification of exposure data and toxicokinetic profile;
3. Evaluation of in vitro toxicity in mammalian cell lines;
4. Examination of potential molecular mechanisms of toxicity derived from in vitro data;
5. Review and analysis of in vivo toxicity data in rodent models, including oral, dermal, and inhalation exposures;
6. Assessment of qualitative evidence on specific and general human health issues linked to triazole fungicide exposure according to the Pesticide Properties DataBase records.

## 2. European Union-Approved Triazole Fungicides

For a fungicide to be approved in the European Union, the applicant must submit a complete dossier, and the active substance must meet the approval criteria laid out in Annex II of Regulation (EC) No 1107/2009. These criteria encompass assessments of human health impacts, environmental fate and behavior, ecotoxicology, and groundwater safety, each supported by specific threshold values or conditions that must be satisfied for approval to be granted. Upon expiry of the approval period, renewal is possible only if the active substance continues to comply with the current approval criteria in force at the time of re-evaluation [6].

A total of 35 triazole fungicides were analyzed with respect to their regulatory status within the European Union (Table 1). In this research, only triazole fungicides that remain approved for use according to current EU regulations were selected as compounds of interest [60].

Of the 35 triazole fungicides analyzed, 10 were found to have an approved status within the EU. These compounds represent the triazole fungicides of interest included in this review (Table 2).

Although these ten triazole fungicides are approved for use within the European Union, they may still pose potential risks to human health through occupational exposure during their production or application, as well as through accidental or environmental exposure. An additional aggravating factor is the potential for improper handling by non-specialized individuals, which may result in accidental direct exposure during application or indirect exposure through environmental contamination. Therefore, understanding the potential toxicological effects of these fungicides on human health is essential.

## 3. Exposure Data and Toxicokinetic Profile of EU-Approved Triazole Fungicides

The human body may come into contact with triazole fungicides through several potential exposure routes, including oral exposure via the ingestion of contaminated food, dermal exposure through direct contact with concentrated formulations during handling, and inhalation of vapors released when these are applied in agriculture. However, the available scientific literature provides relatively limited information addressing and detailing these exposure pathways.

The Pesticide Properties DataBase includes information on parameters such as the Acceptable Daily Intake (ADI), Acute Acceptable Operator Exposure Level (AAOEL), and Acceptable Operator Exposure Level—Systemic (AOEL) [62]. Nevertheless, these data are primarily derived from studies conducted on mammalian models and are not available for all triazole fungicides currently approved for use within the European Union.

Data on ADI and AOEL are reported for all ten triazole fungicides of interest, ranging from 0.004 mg/kg bw/day for TET to 0.036 mg/kg bw/day for PRO in the case of ADI, and from 0.01 mg/kg bw/day for TRI to 0.16 mg/kg bw/day for DIF for AOEL. Data regarding AAOEL are not available for BRO, DIF, PAC, PEN, TEB, and TET; for the remaining compounds, AAOEL values range from 0.01 mg/kg bw/day for MET to 0.2 mg/kg bw/day for PRO [63,64,65,66,67,68,69,70,71,72].

Information on the toxicokinetic profile, encompassing absorption, distribution, metabolism, and excretion (ADME) of EU-approved triazole fungicides is quite limited, both in terms of experimental data and in silico predictions. Such information could not be identified for BRO, MEF and PEN.

Computational predictions using ADMETlab 2.0 and admetSAR 2.0 indicated that DIF has good bioavailability, high intestinal absorption, good oral bioavailability, high plasma protein binding, moderate clearance, and the ability to penetrate the blood–brain barrier [73]. Another in silico study, using FAFDrugs4 and SwissADME, indicated that MET, PAC, TEB, TET, and TRI exhibit good oral bioavailability, can be readily absorbed in the gastrointestinal tract, and are able to reach systemic circulation. These compounds are also predicted to have a moderate ability to penetrate the skin and to cross the blood–brain barrier, and may be metabolized by cytochrome P450 enzymes (CYPs). Additionally, TRI is predicted to be a P-glycoprotein (P-gp) substrate, suggesting potential active efflux both from the gastrointestinal tract back into the lumen and from the brain [74]. TRI was further evaluated, in another study, using ADMETlab 2.0, which predicted high intestinal absorption, moderate clearance, and high plasma protein binding [75].

For PRO, ADME data were derived from in vitro CYP inhibition assays and a physiologically-based pharmacokinetic (PBPK) model. These studies indicated no significant inhibition of CYP2C9, CYP2C19, or CYP3A substrates at dietary exposure levels corresponding to the ADI [76]. Another study indicated that the phase I metabolism of PRO is primarily mediated by CYP2C19 in the liver, based on in vitro experiments using human liver microsomes and recombinant human CYP450 enzymes to convert prothioconazole to its metabolite prothioconazole-desthio [77]. Although in silico data were identified, it should be taken into account that these predictions may differ from experimental results, as has been observed for organophosphates [78].

## 4. Literature Data-Mining and Analysis Workflow

**Figure 1:**
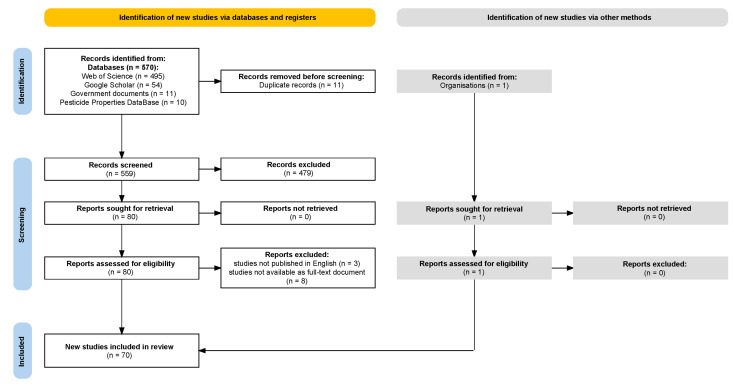
PRISMA diagram illustrating the systematic workflow for data acquisition, screening, eligibility assessment, and final inclusion in the analysis (diagram created using the PRISMA 2020 Flow Diagram Shiny App [80]).

Data mining from the scientific literature regarding the toxicity of triazole fungicides of interest on human health, as well as other relevant information, and the subsequent selection and analysis of these data, were conducted in accordance with PRISMA guidelines (Figure 1). The protocol for this systematic review was registered in Open Science Framework [79], registration DOI:10.17605/OSF.IO/NVZ5X.

The PICO (Population, Intervention/Exposure, Comparator, Outcomes) research question for this systematic review was: What are the toxicological effects of triazole fungicides in in vitro cell lines and in vivo mammalian models, including in vivo endpoints (LD_50_, LC_50_), in vitro endpoints (IC_50_, EC_50_, LC_50_, LOEC), molecular mechanisms of cytotoxicity, and human health issues derived from the Pesticide Properties DataBase?

Following the PICO framework, studies were identified through extensive literature searches across multiple databases, including Web of Science (last search conducted on 12 November 2025), Google Scholar (last consulted on 5 November 2025), and Pesticide Properties DataBase (last searched on 7 November 2025).

The Web of Science (WoS) database [59] was specifically used to retrieve relevant data on the fungicides of interest. This search targeted both in vivo and in vitro studies related to human health, reporting quantitative toxicity measures such as the median lethal dose (LD_50_), median lethal concentration (LC_50_), half-maximal inhibitory concentration (IC_50_), half-maximal effective concentration (EC_50_), or lowest observed effect concentration (LOEC). The following query structure was used, where the generic term “fungicide” was replaced sequentially with the name of each of the ten fungicides of interest in the search strings: TS = (fungicide) AND ((TS = (in vivo) OR TS = (in vitro) OR TS = (human health*)) AND (TS = (LC_50_) OR TS = (IC_50_) OR TS = (EC_50_) OR TS = (LD_50_) OR TS = (LOEC))).

An additional search was performed in Google Scholar (GS) [81], where an exhaustive screening of relevant literature was undertaken. Keywords used in the search string included the names of the ten fungicides of interest combined with terms such as “in vivo”, “rat”, “mouse”, “rabbit”, “*Rattus norvegicus*”, “*Mus musculus*”, “*Oryctolagus cuniculus*”, “LD_50_”, and “LC_50_” for in vivo studies. For in vitro studies, the search terms included each fungicide name together with “in vitro”, “cell”, “cytotoxicity”, “viability”, and “IC_50_”. Depending on the relevance of the search results, articles were screened from the first pages displayed, prioritizing those that reported experimental toxicity data. In addition to peer-reviewed scientific literature, supplementary information sources such as governmental toxicological reports, Safety Data Sheets (SDS) provided by fungicide manufacturers, and records from Pesticide Properties DataBase (PPDB) [62] were also examined.

The screening and selection of studies were performed collaboratively by the review authors, with specific responsibilities assigned to each database. In the Web of Science (WoS) database, one reviewer analyzed the total number of records retrieved using the predefined search strategy and identified those meeting the established inclusion criteria. In Google Scholar (GS), in vitro studies were searched and screened by one reviewer, whereas in vivo studies were independently identified by another reviewer, both following the same eligibility criteria described above. Data retrieved from PPDB were collected by a different author, who extracted both quantitative and qualitative toxicological information relevant to the fungicides of interest. The screening was performed stepwise: titles were first analyzed for the presence of relevant keywords or related content, abstracts of potentially relevant records were then screened, and full texts were examined if they were deemed relevant to the field of interest. No automation tools were used in any stage of the screening or selection process.

Data extraction was performed by a single reviewer per study. Predefined spreadsheets were used to standardize the process. For in vivo studies, the spreadsheet included fields for the fungicide analyzed, toxicological endpoints (LD_50_ or LC_50_), type of exposure, and the organism used. For in vitro studies, the spreadsheet included fields for the fungicide, tested concentrations, cell line, toxicological parameters (IC_50_, LC_50_, EC_50_, or LOEC), and qualitative information on molecular mechanisms of cytotoxicity. For data extracted from PPDB, quantitative fields included Mammals—Acute oral LD_50_, Mammals—Dermal LD_50_, and Mammals—Inhalation LC_50_, while qualitative fields included Specific human health issues and General human health issues. No automation tools were used at any stage of screening or data collection.

The outcomes of interest included quantitative toxicity measures and qualitative information relevant to human health. For in vivo studies, quantitative endpoints were LD_50_ (oral, dermal) and LC_50_ (inhalation), type of exposure, and the organism used. For in vitro studies, endpoints were IC_50_, LC_50_, EC_50_, LOEC, tested concentrations, and cell line. Qualitative outcomes included molecular mechanisms of cytotoxicity (in vitro) and specific or general human health issues (PPDB). For each included study, all data corresponding to the predefined inclusion criteria were extracted and considered, with only clearly reported values used or values that could be standardized or normalized, such as through unit conversion or calculation from reported ratios. No other data were sought.

In total, 570 sources were identified, including scientific articles, 11 governmental reports (GRs), 1 Safety Data Sheet (SDS) from manufacturing companies and 10 reports from PPDB. Of these, 495 scientific articles were retrieved from WoS, and 54 from GS. The sources included 11 duplicates, resulting in 559 studies. Following this process, 479 studies were excluded because, although they were retrieved from WoS using the search phrases, they were not relevant to human health or did not report the quantitative data of interest.

Following the screening stage, 81 studies were selected for detailed analysis. Of these, 11 studies were excluded based on two criteria: studies were not published in English and some studies were not available as full-text documents. No study meeting the inclusion criteria was excluded unless it also met one or more of the exclusion criteria. Consequently, a total of 70 studies were included in this systematic review.

**Figure 2:**
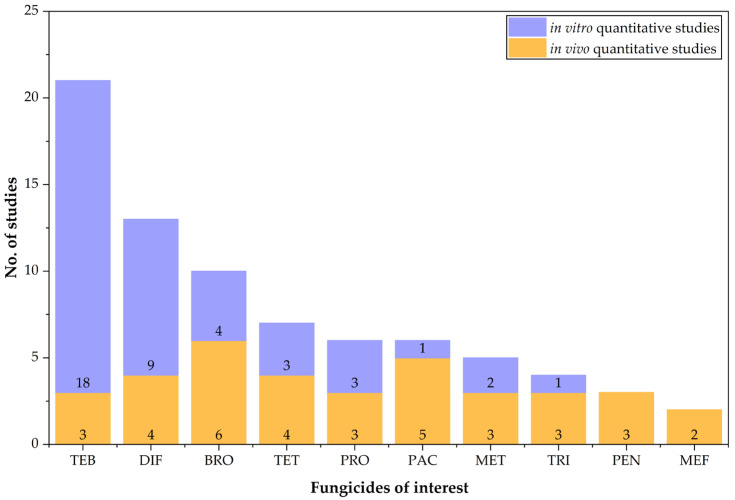
Number of studies analyzed for in vitro (light blue bar) and in vivo (light orange bar) quantitative data of the EU approved triazole fungicides toxicological studies (total counts include duplicate studies shown in both categories).

Of the 70 studies analyzed, 36 provided quantitative data from in vitro experiments on cell lines [82,83,84,85,86,87,88,89,90,91,92,93,94,95,96,97,98,99,100,101,102,103,104,105,106,107,108,109,110,111,112,113,114,115,116,117], while 34 reported quantitative data from in vivo studies in laboratory rodents (this number also includes governmental and company reports, as well as PPDB entries) [63,64,65,66,67,68,69,70,71,72,118,119,120,121,122,123,124,125,126,127,128,129,130,131,132,133,134,135,136,137,138,139,140,141] (Figure 2). Qualitative data were extracted exclusively from these 70 selected studies.

Data analysis involved normalization of measurement units where applicable. For this review, oral and dermal LD_50_ values from mammalian exposure studies were expressed in mg/kg body weight (BW), LC_50_ values from inhalation exposure studies in mg/L, and IC_50_, LC_50_, EC_50_, and LOEC values from in vitro tests in micromolar (µM). Some toxicity endpoints reported as ratios were standardized by converting to their corresponding absolute values to allow comparison across studies. Studies were grouped into in vitro and in vivo categories for synthesis based on the experimental model used. This separation was made to account for differences in biological relevance and data comparability between test systems.

Studies were assigned to syntheses based on endpoint type, exposure route, and experimental model to ensure that only comparable data were grouped for analysis. For each outcome, the effect measures reported in the original studies were used. Values were extracted as presented in the articles, with minimal normalization or standardization applied where necessary. Very few studies reported measures of variability, such as standard deviations; therefore, the extracted data do not include this information.

Quantitative data for both in vivo and in vitro studies were tabulated and provided in Supplementary Material (Tables S1 and S3). Statistical analysis (nonparametric Kruskal-Wallis and Dunn tests), as well as data visualization were conducted using multiple tools, including OriginPro 2025 (OriginLab Corporation, Northampton, MA, USA) for the plotting and analysis of quantitative data, and Cytoscape (version 3.10.4) for the construction and visualization of network relationships between the analyzed fungicides and qualitative toxicity endpoints.

Quantitative data were analyzed and compared descriptively, as most studies did not report standard deviation values, preventing the conduct of group statistics with effect estimate or a meta-analysis. Heterogeneity was explored descriptively through comparative analysis of subgroups, as standard deviation values were largely missing and substantial differences existed in how outcomes were reported across studies. The risk of bias for each of the results was further examined using ToxRTool [142], by one reviewer. Risk of bias due to missing results was considered descriptively, as many studies reported only threshold values or lacked sufficient statistical information, preventing formal quantitative assessment. Formal certainty assessment (e.g., GRADE) was not performed due to high heterogeneity, different in vitro cell lines with few studies per fungicide, and sparse or threshold-based data in in vivo studies per exposure type, preventing reliable grading of confidence. Sensitivity analyses were not performed due to the limited number of comparable studies and the heterogeneity of the available data.

## 5. In Vitro Toxicity of EU-Approved Triazole Fungicides in Mammalian Cell Lines

**Figure 3:**
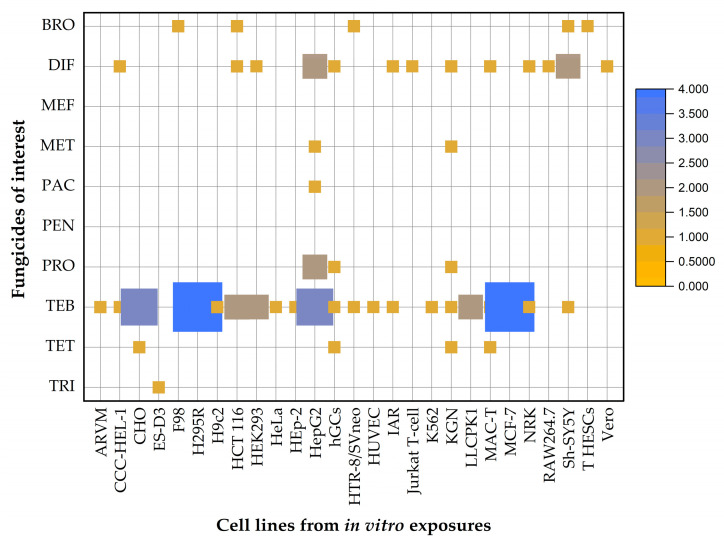
Matrix bubble plot depicting the number and categories of cell lines reported for each fungicide of interest. Larger and more blue bubbles represent a higher number of studies for the corresponding cell line and fungicide, while orange indicates fewer studies. The scale reflects study count both by color and bubble size. The fungicides included in the plot are: Bromuconazole (BRO), Difenoconazole (DIF), Mefentrifluconazole (MEF), Metconazole (MET), Paclobutrazol (PAC), Penconazole (PEN), Prothioconazole (PRO), Tebuconazole (TEB), Tetraconazole (TET), and Triticonazole (TRI). Cell lines represented in the plot are: adult rat ventricular myocytes (ARVM), human hepatocytes (CCC-HEL-1), Chinese hamster ovary cells (CHO), mouse embryonic multipotent stem cell line (ES-D3), rat glial-like cells (F98), human adrenocortical cell line (H295R), rat heart myoblast cells (H9c2), human colon cancer cell line (HCT 116), human renal cells (HEK293), human cervical carcinoma derived immortalized cell line (HeLa), human HeLa-contaminated laryngeal carcinoma-derived epithelial cells (HEp-2), human hepatocellular carcinoma-derived epithelial cells (HepG2), human primary granulosa cells (hGCs), human trophoblast immortalized cell line (HTR-8/SVneo), human endothelial cell line (HUVEC), rat liver epithelial cells (IAR), human T lymphocyte cell line (Jurkat T-cell), human leukemia-derived lymphoblast cells (K562), human granulosa line (KGN), pig kidney-derived epithelial cells (LLCPK1), cattle mammary alveolar epithelial cell (MAC-T), human breast cancer cell line (MCF-7), rat kidney cell line (NRK), mouse macrophage cell line (RAW264.7), human neuroblastoma cell line (SH-SY5Y), human uterine immortalized fibroblasts (T HESCs), African green monkey kidney-derived epithelial cells (Vero).

The analysis of the selected articles allowed the identification of quantitative data for all fungicides of interest, with the exception of MEF and PEN, for which data gaps were observed. The in vitro data were obtained across a wide range of cell lines, including cancerous, transformed, and immortalized lines, derived from human and other mammalian species. The cell lines with the most studies and data across multiple fungicides were HepG2, KGN, and hGCs. In contrast, CHO, H295R, HepG2, and MCF-7 were the cell lines with the most data available for the same fungicide, specifically TEB (Figure 3).

**Figure 4:**
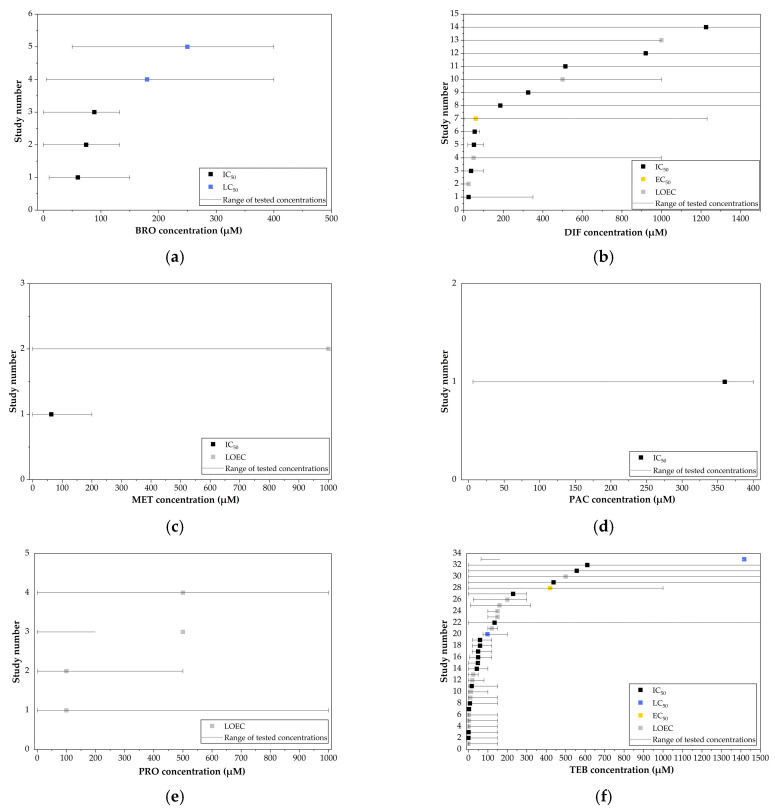

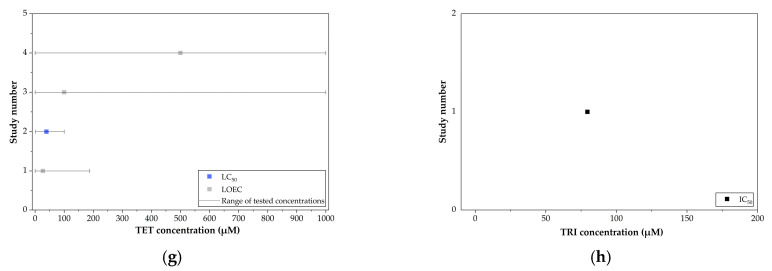
Forest-like plot of in vitro toxicity values (IC_50_, LC_50_, EC_50_, LOEC) for EU-Approved Triazole Fungicides with Tested Concentration Ranges: (**a**) BRO, (**b**) DIF, (**c**) MET, (**d**) PAC, (**e**) PRO, (**f**) TEB, (**g**) TET and (**h**) TRI. The values shown in this figure correspond to data presented in Table S1 of the Supplementary Material.

The in vitro data included IC_50_, LC_50_, EC_50_, and LOEC values, which were used to generate forest-like plots, with the error bars representing the tested concentration ranges. DIF and TEB had the largest number of data points, whereas PAC and TRI each had only a single reported value (Figure 4).

The combined median values of the analyzed toxicological indices indicated that TET was the most cytotoxic, exhibiting the lowest value, whereas DIF was the least cytotoxic, with the highest value. The overall toxicity ranking was TET > MET > TRI > BRO > TEB > DIF. No comparable results could be identified for MEF, PEN, and PRO. In the in vitro studies, heterogeneity was primarily driven by the animal strain used, followed by inconsistencies in reporting formats (LOEC values versus toxicological indices).

Risk of bias analysis using the ToxRTool software (2009 version) [142] showed that only a limited number of studies met the criteria for classification into Class 1 (Supplementary Table S1). These included LOEC data for PRO in HepG2 cells, TEB in HepG2 cells, and TEB in SH-SY5Y cells, as well as toxicological indices for TEB in HeLa and HepG2 cells and TRI in ES-D3 cells. This indicates that only a small subset of the available evidence met high-quality methodological standards, suggesting that overall confidence in the body of evidence is constrained by variable study quality and potential sources of bias in the remaining studies. Potential risk of bias from missing results in in vitro studies was evaluated descriptively, but the diversity of endpoints and cell lines precluded statistical assessment.

## 6. Molecular Mechanisms Underlying In Vitro Toxicity of EU-Approved Triazole Fungicides

**Figure 5:**
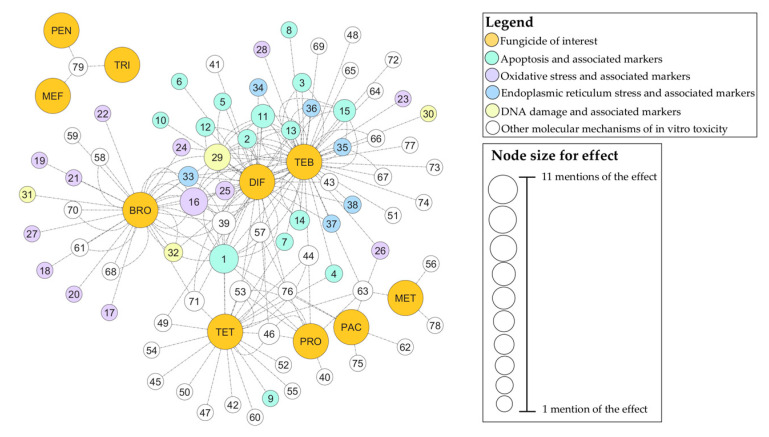
Cytoscape-generated network of EU-approved triazole fungicides and associated molecular mechanisms of in vitro cytotoxicity. Numbers shown on network nodes correspond to specific molecular-level effects, detailed in Table S2 of the Supplementary Material.

Most of the analyzed in vitro studies also assessed the mechanisms of toxicity by evaluating specific molecular markers. Among these molecular mechanisms, cellular apoptosis was the most frequently observed across the largest number of fungicides and studies, followed by oxidative stress. The fungicides for which the molecular mechanisms of cytotoxicity were most extensively investigated were DIF and TEB, whereas MEF, PEN, and TRI had no data regarding these aspects (Figure 5).

Regarding apoptosis, the most frequently assessed markers were the Bax/Bcl-2 ratio, reduction of mitochondrial membrane potential, and induction of poly (ADP-ribose) polymerase (PARP) cleavage. In addition, the expression levels of caspases 3, 7, 8, and 9 were also evaluated. For oxidative stress, reactive oxygen species (ROS) level was the most commonly measured parameter, alongside malondialdehyde (MDA), catalase (CAT), superoxid dismutase (SOD), glutathione peroxidase (GPX), and glutathione-S-transferase (GST). For endoplasmic reticulum (ER) stress, parameters included the expression of activating transcription factor 4 (ATF4), binding-immunoglobulin protein/glucose regulated protein78 (Bip/GRP78), C/EBP homologous protein (CHOP), and endoplasmic oxidoreductin-1-like (ERO1-Lα).

## 7. In Vivo Toxicity of EU-Approved Triazole Fungicides in Rodent Models

**Figure 6:**
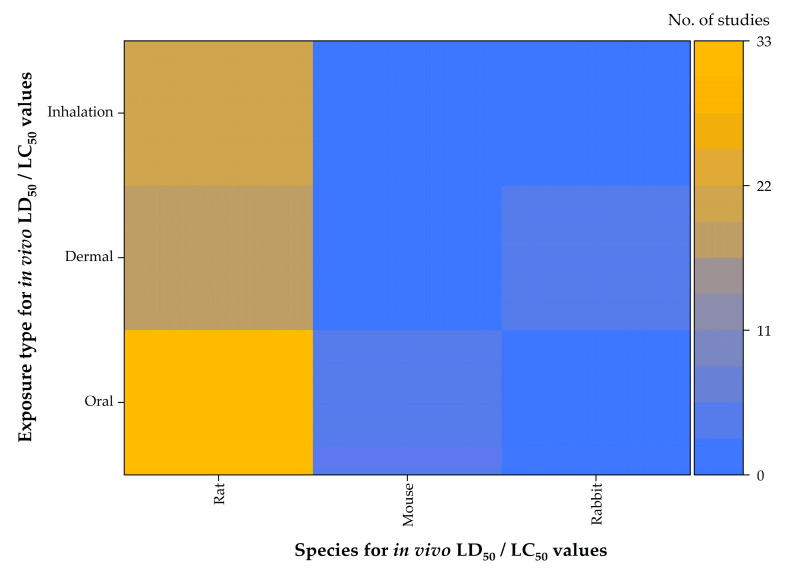
Heatmap visualization of in vivo LD_50_/LC_50_ values for the analyzed triazole fungicides, organized by exposure type (oral, dermal, inhalation) and test species (rat, mouse, rabbit).

The analysis of the selected articles enabled the identification of quantitative in vivo toxicity data for all fungicides of interest, with the exception of MEF regarding oral exposure, for which a data gap was observed. The available data included LD_50_ values for oral and dermal exposures and LC_50_ values for inhalation studies. Most of the results were obtained from experiments conducted on rats, while a smaller number of studies involved mice (oral exposure) and rabbits (inhalation exposure) (Figure 6). For the in vivo data, only a limited number of exact categorical values were identified; therefore, the analysis and visualization also included data reported as being greater than a specific threshold value.

**Figure 7:**
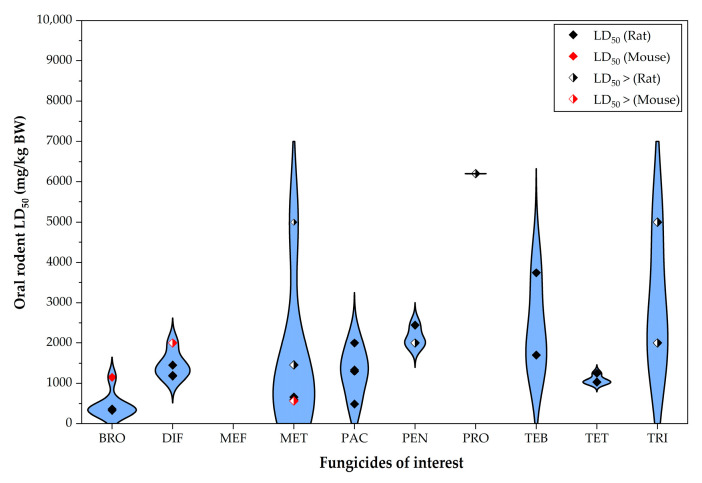
Violin plot illustrating the distribution of oral LD_50_ values for the triazole fungicides of interest. The values shown in this figure correspond to data presented in Table S3 of the Supplementary Material.

The oral LD_50_ values indicated that the most toxic fungicides for rodents were BRO, followed by TET, whereas the least toxic were PRO and TRI. BRO also had the highest number of studies reporting quantitative oral toxicity data. For fungicides with available data in both rats and mice, some difference between the LD_50_ values of the two species were observed (Figure 7).

**Figure 8:**
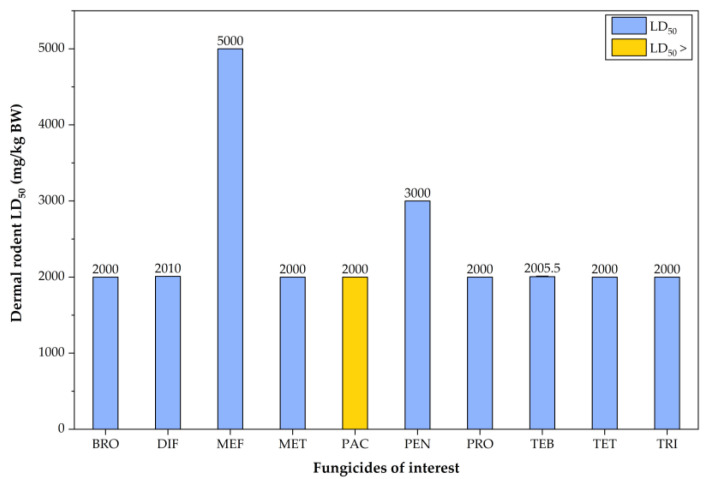
Column plot illustrating the distribution of dermal LD_50_ values for the triazole fungicides of interest. The values shown in this figure correspond to data presented in Table S3 of the Supplementary Material.

The dermal LD_50_ values were relatively similar among the analyzed fungicides, except for MEF and PEN, which showed higher LD_50_ values, indicating lower dermal toxicity compared to the others (Figure 8).

**Figure 9:**
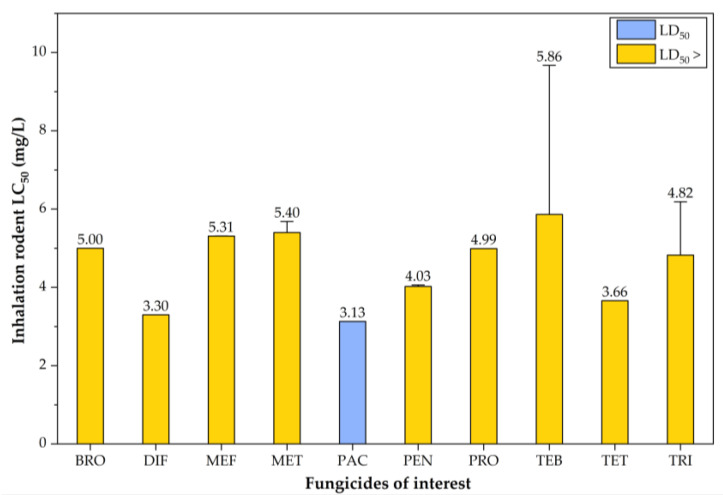
Column plot illustrating the distribution of inhalation LC_50_ values for the triazole fungicides of interest. The values shown in this figure correspond to data presented in Table S3 of the Supplementary Material.

The LC_50_ values for inhalation exposure indicated higher inhalation toxicity for PAC, DIF, and TET, with recorded values of 3.13, 3.30, and 3.66 mg/L, respectively (Figure 9).

The in vivo data indicated that BRO, DIF, PAC, and TET exhibit higher toxicity in rodents, whereas MEF, PEN, PRO, and TRI were associated with lower toxicity levels. The heterogeneity of the in vivo data was driven by differences in the reporting of toxicological indices—specifically the use of exact values versus threshold (greater than) values—as well as variation in the species used across studies.

Risk of bias assessment showed that all in vivo studies were classified as Class 3, indicating limited methodological detail and uncertainty regarding study quality. Risk of bias from missing results in in vivo studies was assessed descriptively; sparse and threshold-based data prevented statistical analysis. Statistical analysis further showed that significant differences were identified only within the in vivo dataset, specifically for oral toxicity values in rats. The analysis included only exact reported values, excluding threshold-type data. A Kruskal–Wallis test revealed statistically significant differences between fungicides, and post-hoc Dunn tests indicated significant differences between BRO and DIF, and between BRO and TEB, with BRO demonstrating higher toxicity in both comparisons.

## 8. Qualitative Human Health Effects Associated with EU-Approved Triazole Fungicide Exposure

**Figure 10:**
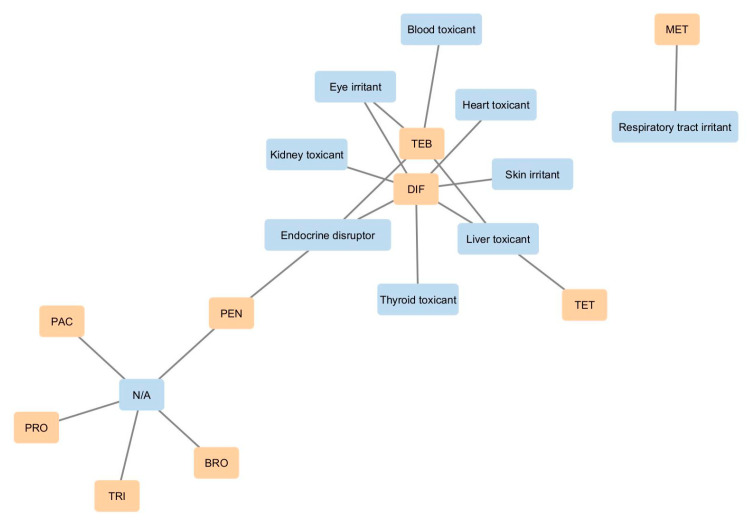
Cytoscape-generated network of EU-approved triazole fungicides and associated human health effects identified from PPDB database. Nodes representing the fungicides of interest are colored orange, while those corresponding to specific human health issues are shown in blue (N/A – not available).

Network analysis of the PPDB data regarding general and specific human health effects associated with the EU-approved triazole fungicides revealed marked differences in data availability. DIF and TEB were the most extensively characterized, with DIF linked to endocrine disruption, thyroid toxicity, liver toxicity, cardiotoxicity, and nephrotoxicity, and TEB associated with hematotoxicity, liver toxicity, eye irritation, and endocrine disruption. MET was reported only as a respiratory tract irritant, while PEN was noted solely for endocrine disruption. No specific human health data were available for BRO, PAC, PRO, or TRI (Figure 10).

This analysis highlights substantial gaps in human health hazard data for several EU-approved triazole fungicides and underscores the need for targeted toxicological investigation, particularly for those compounds with little to no recorded information.

## 9. Discussion

A previous review on the genotoxic effects of triazole fungicides [55] reported the potential of these compounds to induce DNA damage and oxidative stress in vivo, consistent with the patterns observed in our analysis of in vitro data. In addition to these findings, our review addresses EU-approved triazole fungicides and integrates evidence from in vitro cytotoxicity, mechanistic studies, and toxicokinetic data, alongside in vivo results. This complementary approach provides a broader perspective on multiple toxicity domains and potential human health effects, extending the insights reported in the previous review, which focused specifically on in vivo genotoxicity in mammals.

Another study investigating the impact of triazole fungicides on female fertility and embryonic development [57] reported that these compounds may inhibit or interfere with enzymes such as CYP19A1, induce oxidative stress, and trigger apoptosis, while a second study on chiral triazole fungicides in mammalian models [58] highlighted oxidative stress and endocrine disruption, which further supports the patterns observed in our analysis of in vitro studies.

The triazole fungicides analyzed in this paper were observed to induce oxidative stress in cells, consistent with previous findings for propiconazole [143]. Another effect observed for triazole fungicides in our review was the induction of apoptosis in in vitro studies, consistent with previous findings for other fungicides [144]. A study investigating the neurotoxicity of certain fungicides in primary cultured mouse cortical neurons [145] reported depolarization of the mitochondrial membrane potential, a mechanism of toxicity also observed for triazole fungicides in our review.

A study identified an LD_50_ value for acute oral exposure to phenylpyrrole in rats [146], reporting a value greater than 5000 mg/kg BW, which is consistent with some of the results observed for triazole fungicides in our review.

These aspects discussed above, including key outcomes reported for triazole and other fungicides in the literature, are further summarized in Table S4 of the Supplementary Materials, providing a visual overview of how these findings align with or differ from the results of the current study on EU-approved triazoles.

The available literature indicates that this review aligns with observed patterns; however, heterogeneity in data, reporting formats, and methodologies—both within this review and in other studies—prevents a more direct and detailed comparison.

The toxicological and mechanistic data compiled in this review are potentially relevant for regulatory evaluation and risk assessment of EU-approved triazole fungicides, as they provide additional evidence on human health effects and toxicity pathways. According to Regulation (EC) No 1107/2009 [6], approval dossiers must include, among other relevant data, scientific peer-reviewed literature published within the last ten years addressing effects on human health, the environment, and non-target species. In this context, the present review compiles in vitro, in vivo, mechanistic and toxicokinetic data, providing complementary evidence to support a comprehensive assessment of triazole fungicides in the EU.

The review reveals notable gaps in the available data on EU-approved triazole fungicides, including limited in vitro, in vivo, and toxicokinetic information for several compounds. Mechanistic and human health-related evidence is incomplete, restricting comprehensive risk assessment and comparison across all fungicides. These limitations, combined with the widespread use of triazoles, underscore the need for further research and monitoring to support safe and sustainable application. Addressing these gaps will inform risk management and regulatory practices to better protect human health within the One Health framework. Moreover, future research on environmental impacts and ecotoxicity of triazole fungicides needs to be further explored to provide a more comprehensive One Health perspective.

## 10. Conclusions

This systematic review provides an integrated evaluation of the toxicological evidence related to ten triazole fungicides currently approved for use within the European Union. Both in vitro and in vivo data indicate that these compounds exhibit varying degrees of toxicity, with bromuconazole, difenoconazole, paclobutrazol, and tetraconazole showing comparatively higher toxic potential in rodent models, while mefentrifluconazole, penconazole, prothioconazole, and triticonazole demonstrated lower acute toxicity.

The in vitro findings revealed consistent cytotoxic effects across a wide range of mammalian cell lines, with tetraconazole emerging as the most cytotoxic compound based on combined quantitative indices. The most frequently investigated mechanisms of toxicity included cellular apoptosis and oxidative stress, followed by endoplasmic reticulum stress and genotoxicity, suggesting that mitochondrial dysfunction and redox imbalance play central roles in triazole-induced cytotoxicity.

Toxicokinetic information, encompassing absorption, distribution, metabolism, and excretion (ADME), remains limited and often derived from in silico predictions rather than experimental data. Available evidence points to good oral absorption, high plasma protein binding, and potential interactions with cytochrome P450 enzymes for several fungicides, underlining the need for more comprehensive pharmacokinetic and metabolic studies.

Qualitative assessments based on the PPDB database highlighted associations between some triazole fungicides and specific human health concerns, such as developmental toxicity, as well as general systemic effects. However, data gaps persist, particularly regarding chronic exposure and cumulative effects.

Overall, this review emphasizes the importance of continued toxicological and mechanistic investigation of EU-approved triazole fungicides to refine human health risk assessments, ensure regulatory safety, and guide the development of less hazardous alternatives.

This study provides an important contribution to the One Health framework regarding triazole fungicides, with a focus on their effects on human health, and highlights the need to continue the research to include environmental and ecotoxicological aspects.

## Figures and Tables

**Table 1:** Triazole fungicides and their regulatory status within the European Union (N/A-not available).

Triazole Fungicide	Status of Approval	Approval Expiration Date
Azaconazole	Not approved	N/A
Bitertanol	Not approved	29 August 2013
Bromuconazole	Approved	30 April 2027
Cyproconazole	Not approved	31 May 2021
Difenoconazole	Approved	15 March 2026
Diniconazole	Not approved	N/A
Epoxiconazole	Not approved	30 April 2020
Etaconazole	Not approved	N/A
Fenbuconazole	Not approved	30 April 2021
Fluconazole	N/A	N/A
Fluotrimazole	N/A	N/A
Fluoxytioconazole	N/A	N/A
Fluquinconazole	Not approved	31 December 2021
Flusilazole	Not approved	N/A
Flutriafol	Not approved	31 May 2021
Furconazole	Not approved	N/A
Hexaconazole	Not approved	N/A
Imibenconazole	Not approved	N/A
Ipconazole	Not approved	31 May 2023
Ipfentrifluconazole	N/A	N/A
Mefentrifluconazole	Approved	20 March 2029
Metconazole	Approved	31 August 2031
Myclobutanil	Not approved	31 May 2021
Paclobutrazol	Approved	31 August 2026
Penconazole	Approved	15 October 2026
Propiconazole	Not approved	19 December 2018
Prothioconazole	Approved	31 March 2027
Quinconazole	N/A	N/A
Simeconazole	Not approved	N/A
Tebuconazole	Approved	15 August 2026
Tetraconazole	Approved	31 March 2027
Triadimefon	Not approved	N/A
Triadimenol	Not approved	31 August 2019
Tricyclazole	Not approved	N/A
Triticonazole	Approved	31 January 2027

**Table 2:** Molecular weight and 2D chemical structures of the EU-approved triazole fungicides (MW = molecular weight; data retrieved from PubChem [61]).

Bromuconazole (BRO)	Difenoconazole (DIF)
MW: 377.1 g/mol	MW: 406.3 g/mol
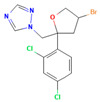	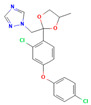
**Mefentrifluconazole (MEF)**	**Metconazole (MET)**
MW: 397.8 g/mol	MW: 319.8 g/mol
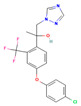	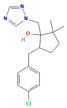
**Paclobutrazol (PAC)**	**Penconazole (PEN)**
MW: 293.79 g/mol	MW: 284.18 g/mol
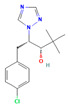	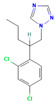
**Prothioconazole (PRO)**	**Tebuconazole (TEB)**
MW: 344.3 g/mol	MW: 307.82 g/mol
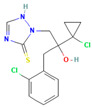	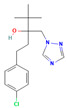
**Tetraconazole (TET)**	**Triticonazole (TRI)**
MW: 372.14 g/mol	MW: 317.8 g/mol
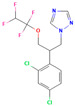	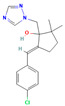

## Data Availability

No new data were created or analyzed in this study.

## Supplementary Materials

The following supporting information can be downloaded at https://www.mdpi.com/article/10.3390/jox15060208/s1. Table S1: In vitro toxicity data for the EU-approved triazole fungicides of interest. Data highlighted in bold are classified as Category 1 according to the ToxRTool evaluation from the risk-of-bias analysis; Table S2: Assigned numbers for molecular mechanisms of in vitro cytotoxicity for Cytoscape network diagram; Table S3: In vivo toxicity data for the EU-approved triazole fungicides of interest; Table S4: Comparative summary of key outcomes from literature-reported fungicide studies, including triazoles and other fungicides, for contextualization with findings from the current study on selected EU-approved triazole in vitro and in vivo toxicity. References [55,56,57,58], [63,64,65,66,67,68,69,70,71,72], [82,83,84,85,86,87,88,89,90,91,92,93,94,95,96,97,98,99,100,101,102,103,104,105,106,107,108,109,110,111,112,113,114,115,116,117,118,119,120,121,122,123,124,125,126,127,128,129,130,131,132,133,134,135,136,137,138,139,140,141], and [143,144,145,146] are cited in the Supplementary Materials.
