# Supplementary material for: A Systematic Review on the Toxicology of European Union-Approved Triazole Fungicides in Cell Lines and Mammalian Models
Source: J Xenobiot. 2025 Dec 5;15(6):208. doi: 10.3390/jox15060208 (PMC12733837; doi:10.3390/jox15060208)
Supplement: Supplementary file 1 [file jox-15-00208-s001.zip › jox-4008954-supplementary.pdf]

Journal of Xenobiotics

Supplementary Materials: A Systematic Review on the Toxicology of European Union-Approved Triazole Fungicides in Cell Lines and Mammalian Models

Constantina-Bianca Vulpe, Adina-Daniela Iachimov-Datcu, Andrijana Pujicic and Bianca-Vanesa Agachi

Table S1. In vitro toxicity data for the EU-approved triazole fungicides of interest. Data highlighted in bold are classified as Category 1 according to the ToxRTool evaluation from the risk-of-bias analysis.

Fungicide	Cell line	TC (μM)	TP	VTP (μM)	Reference
Bromuconazole	F98	10-150	IC_{50}	60	[1]
Bromuconazole	HCT 116	5-400	IC_{50}	180	[2]
Bromuconazole	HTR-8/SVneo	0-133	LC_{50}	74.4	[3]
Bromuconazole	Sh-SY5Y	50-400	IC_{50}	250	[4]
Bromuconazole	T HESCs	0-133	LC_{50}	88.6	[3]
Difenoconazole	CCC-HEL-1	0-6153	IC_{50}	921	[5]
Difenoconazole	HCT 116	0-2461	IC_{50}	1.55×10^4	[6]
Difenoconazole	HEK293T	0-6153	IC_{50}	514	[5]
Difenoconazole	HepG2	0-350	IC_{50}	24.7	[7]
Difenoconazole	HepG2	N/A	LOEC	25	[8]
Difenoconazole	hGCs	0.001-1000	LOEC	500	[9]
Difenoconazole	IAR	0-6153	IC_{50}	326	[5]
Difenoconazole	Jurkat T-cell	1.231-1231	EC_{50}	60.7	[7]
Difenoconazole	KGN	0.001-1000	LOEC	1×10^3	[9]
Difenoconazole	MAC-T	0-1000	LOEC	50	[10]
Difenoconazole	NRK	0-6153	IC_{50}	1.23×10^3	[5]
Difenoconazole	RAW264.7	0-100	IC_{50}	37.1	[11]
Difenoconazole	SH-SY5Y	20-100	IC_{50}	51.7	[12]
Difenoconazole	SH-SY5Y	0-80	IC_{50}	55.4	[13]
Difenoconazole	Vero	0-2461	IC_{50}	185	[6]
Mefentrifluconazole	N/A	N/A	N/A	N/A	N/A
Metconazole	HepG2	0-200	IC_{50}	63	[14]
Metconazole	KGN	0.001-1000	LOEC	1×10^3	[9]
Paclobutrazol	HepG2	6.25-400	IC_{50}	360	[15]
Penconazole	N/A	N/A	N/A	N/A	N/A
Prothioconazole	HepG2	1-500	LOEC	100	[16]
**Prothioconazole**	**HepG2**	**0-200**	**LOEC**	**500**	**[17]**
Prothioconazole	hGCs	0.001-1000	LOEC	100	[9]
Prothioconazole	KGN	0.001-1000	LOEC	500	[9]
Tebuconazole	ARVM	20-120	IC_{50}	60	[18]
Tebuconazole	CCC-HEL-1	0-8121	IC_{50}	438	[5]
Tebuconazole	CHO	0.001-150	IC_{50}	8.1	[19]
Tebuconazole	CHO	75-200	LC_{50}	98.1	[20]
Tebuconazole	CHO	0.001-150	LOEC	3.1	[19]
Tebuconazole	H295R	0.001-150	IC_{50}	0.5	[19]
Tebuconazole	H295R	0.001-150	IC_{50}	1.2	[19]
Tebuconazole	H295R	0.001-150	LOEC	0.1	[19]
Tebuconazole	H295R	0.001-150	LOEC	3	[19]
Tebuconazole	H9c2	20-120	IC_{50}	60	[18]
Tebuconazole	HCT 116	5-120	IC_{50}	50	[21]
Tebuconazole	HCT 116	20-120	IC_{50}	50	[22]

Tebuconazole	HEK293	100-150	LOEC	120	[23]
Tebuconazole	HEK293T	0-8121	IC_{50}	135	[5]
**Tebuconazole**	**HeLa**	**0.025-3.249**	**IC_{50}**	**3.25**	**[24]**
Tebuconazole	HEp-2	64.97-162-433	LC_{50}	1.42×10^3	[25]
**Tebuconazole**	**HepG2**	**1-1000**	**EC_{50}**	**420**	**[26]**
**Tebuconazole**	**HepG2**	**0.5-50**	**LOEC**	**25**	**[27]**
Tebuconazole	HepG2	10-320	LOEC	160	[28]
Tebuconazole	hGCs	0-100	LOEC	12.5	[29]
Tebuconazole	HTR-8/Svneo	0-80	LOEC	20	[30]
Tebuconazole	HUVEC	25000-200000	IC_{50}	25.8×10^4	[31]
Tebuconazole	IAR	0-8121	IC_{50}	611	[5]
Tebuconazole	K562	0-100	IC_{50}	43.5	[32]
Tebuconazole	KGN	0-10000	LOEC	500	[29]
Tebuconazole	LLCPK1	100-150	LOEC	150	[23]
Tebuconazole	LLCPK1	100-150	LOEC	150	[23]
Tebuconazole	MAC-T	0-300	IC_{50}	230	[33]
Tebuconazole	MCF-7	0.001-150	IC_{50}	17	[19]
Tebuconazole	MCF-7	0.001-150	IC_{50}	49	[19]
Tebuconazole	MCF-7	0.001-150	LOEC	1.6	[19]
Tebuconazole	MCF-7	0.001-150	LOEC	10	[19]
Tebuconazole	NRK	0-8121	IC_{50}	557	[5]
**Tebuconazole**	**SH-SY5Y**	**25-300**	**LOEC**	**200**	**[34]**
Tetraconazole	CHO	1-100	LC_{50}	39.2	[20]
Tetraconazole	hGCs	0.001-1000	LOEC	500	[9]
Tetraconazole	KGN	0.001-1000	LOEC	100	[9]
Tetraconazole	MAC-T	0-188	LOEC	26.9	[35]
**Triticonazole**	**ES-D3**	**N/A**	**IC_{50}**	**79.5**	**[36]**

TC = tested concentration; TP = toxicological parameters; VTP = value of toxicological parameters.; N/A = data not available.

Table S2. Assigned numerical codes representing molecular mechanisms of *in vitro* cytotoxicity, used to generate the Cytoscape network diagram shown in Figure 5. Each number corresponds to a specific mechanism identified in the in vitro studies included in this review.

Molecular mechanism of in vitro cytotoxicity	Assigned number
Induced apoptosis	1
Activated Caspase-3	2
Activated Caspase-9	3
Increased Bax expression	4
Increased Caspase-3 expression	5
Increased Caspase-7 expression	6
Increased cleaved Caspase-3 expression	7
Increased cleaved Caspase-8 expression	8
Increased Cyt C expression	9
Increased PARP expression	10
Increased the Bax/Bcl-2 ratio	11
Induced collapse of mitochondrial membrane potential	12
Induced Cyt C release	13
Induced PARP cleavage	14
Reduced mitochondrial membrane potential	15
Increased ROS level	16
Disrupted CAT activity	17
Disrupted SOD activity	18
Enhanced CAT activity	19

Enhanced GPX activity	20
Enhanced GST activity	21
Enhanced SOD activity	22
Increased CAT activity	23
Increased lipid peroxidation level	24
Increased MDA level	25
Increased oxidative stress	26
Increased PC level	27
Increased SOD activity	28
Induced DNA damage	29
Induced genotoxicity	30
Induced nuclear condensation	31
Inhibited DNA synthesis	32
Induced ER stress	33
Increased ATF4 expression	34
Increased Bip/GRP78 expression	35
Increased CHOP expression	36
Increased ERO1-Lα expression	37
Increased PDI expression	38
Arested cell cycle in G0/G1 phase	39
Activated AHR	40
Decreased AHR expression	41
Decreased AKT signaling pathway activity	42
Decreased CYP11A1 expression	43
Decreased CYP19A1 mRNA expression	44
Decreased CYP51 expression	45
Decreased E2 level	46
Decreased ERK1/2 signaling pathway activity	47
Decreased expression of milk-protein-synthesis-related genes	48
Decreased HSD3B expression	49
Decreased JNK signaling pathway activity	50
Decreased MTP level	51
Decreased P38 signaling pathway activity	52
Decreased Pg level	53
Decreased proliferation-related protein CCND1	54
Decreased proliferation-related protein PCNA	55
Decreased STAR expression	56
Decreased STAR mRNA expression	57
Disregulated MAPK signaling	58
Disrupted cell cytoskeleton	59
Disrupted mitochondrial Ca^{2+} homeostasis	60
Disrupted mitochondrial function	61
Impaired autophagy	62
Increased AHR mRNA expression	63
Increased expression of lipid uptake and oxidation-related markers	64
Increased nuclear translocation of PPARs	65
Increased p21 protein level	66
Increased p53 protein level	67
Induced inflammation	68
Induced lipid accumulation	69
Inhibited cell migration	70
Inhibited cell proliferation	71
Inhibited MATE1	72
Inhibited MATE2-K	73
Inhibited OCTN2	74

Involvement of AMPK/mTOR signaling pathway	75
Reduced cell viability	76
Reduced steroid secretion	77
Decreased CYP19A1 expression	78
N/A	79

Table S3. *In vivo* toxicity data for the EU-approved triazole fungicides of interest

Fungicide	Exposure type	TP	VTP	VTP M.U.	Reference
Bromuconazole	Dermal	LD_{50}	2×10^3	mg/kg BW	[37]
Bromuconazole	Dermal	LD_{50}	$> 2 \times 10^3$	mg/kg BW	[38]
Bromuconazole	Dermal	LD_{50}	$> 2 \times 10^3$	mg/kg BW	[39]
Bromuconazole	Inhalation	LC_{50}	> 5	mg/L	[38]
Bromuconazole	Inhalation	LC_{50}	> 5	mg/L	[39]
Bromuconazole	Inhalation	LC_{50}	> 5	mg/L	[37]
Bromuconazole	Oral	LD_{50}	328	mg/kg BW	[38]
Bromuconazole	Oral	LD_{50}	328	mg/kg BW	[40]
Bromuconazole	Oral	LD_{50}	328	mg/kg BW	[39]
Bromuconazole	Oral	LD_{50}	328	mg/kg BW	[37]
Bromuconazole	Oral	LD_{50}	365	mg/kg BW	[41]
Bromuconazole	Oral	LD_{50}	365	mg/kg BW	[42]
Bromuconazole	Oral	LD_{50}	1.15×10^3	mg/kg BW	[41]
Difenoconazole	Dermal	LD_{50}	20.1×10^2	mg/kg BW	[43]
Difenoconazole	Dermal	LD_{50}	$< 20.1 \times 10^2$	mg/kg BW	[44]
Difenoconazole	Inhalation	LC_{50}	> 3.3	mg/L	[44]
Difenoconazole	Inhalation	LC_{50}	> 3.3	mg/L	[43]
Difenoconazole	Oral	LD_{50}	1.18×10^3	mg/kg BW	[45]
Difenoconazole	Oral	LD_{50}	1.2×10^3	mg/kg BW	[46]
Difenoconazole	Oral	LD_{50}	1.45×10^3	mg/kg BW	[44]
Difenoconazole	Oral	LD_{50}	1.45×10^3	mg/kg BW	[43]
Difenoconazole	Oral	LD_{50}	$> 2 \times 10^3$	mg/kg BW	[44]
Mefentrifluconazole	Dermal	LD_{50}	5×10^3	mg/kg BW	[47]
Mefentrifluconazole	Dermal	LD_{50}	$> 5 \times 10^3$	mg/kg BW	[48]
Mefentrifluconazole	Inhalation	LC_{50}	> 5.3	mg/L	[47]
Mefentrifluconazole	Inhalation	LC_{50}	> 5.3	mg/L	[48]
Mefentrifluconazole	Oral	LD_{50}	$> 2 \times 10^3$	mg/kg BW	[48]
Mefentrifluconazole	Oral	LD_{50}	$> 2 \times 10^3$	mg/kg BW	[47]
Metconazole	Dermal	LD_{50}	2×10^3	mg/kg BW	[49]
Metconazole	Dermal	LD_{50}	$> 2 \times 10^3$	mg/kg BW	[50]
Metconazole	Dermal	LD_{50}	$> 2 \times 10^3$	mg/kg BW	[50]
Metconazole	Inhalation	LC_{50}	5.6	mg/L	[50]
Metconazole	Inhalation	LC_{50}	> 5.2	mg/L	[49]
Metconazole	Oral	LD_{50}	595	mg/kg BW	[49]
Metconazole	Oral	LD_{50}	660	mg/kg BW	[51]
Metconazole	Oral	LD_{50}	$> 1.46 \times 10^3$	mg/kg BW	[50]
Metconazole	Oral	LD_{50}	$> 5 \times 10^3$	mg/kg BW	[50]
Metconazole	Oral	LD_{50}	> 566	mg/kg BW	[50]
Metconazole	Oral	LD_{50}	> 566	mg/kg BW	[50]
Paclobutrazol	Dermal	LD_{50}	$> 2 \times 10^3$	mg/kg BW	[52]
Paclobutrazol	Dermal	LD_{50}	$> 2 \times 10^3$	mg/kg BW	[53]
Paclobutrazol	Inhalation	LC_{50}	3.1	mg/L	[53]
Paclobutrazol	Inhalation	LC_{50}	3.1	mg/L	[52]
Paclobutrazol	Oral	LD_{50}	490	mg/kg BW	[54]

Paclobutrazol	Oral	LD_{50}	1.3×10^3	mg/kg BW	[55]
Paclobutrazol	Oral	LD_{50}	1.34×10^3	mg/kg BW	[52]
Paclobutrazol	Oral	LD_{50}	2×10^3	mg/kg BW	[56]
Paclobutrazol	Oral	LD_{50}	1.34×10^3	mg/kg BW	[53]
Penconazole	Dermal	LD_{50}	3×10^3	mg/kg BW	[57]
Penconazole	Dermal	LD_{50}	$> 3 \times 10^3$	mg/kg BW	[58]
Penconazole	Inhalation	LC_{50}	> 4	mg/L	[58]
Penconazole	Inhalation	LC_{50}	> 4.1	mg/L	[57]
Penconazole	Oral	LD_{50}	2.44×10^3	mg/kg BW	[59]
Penconazole	Oral	LD_{50}	$> 2 \times 10^3$	mg/kg BW	[58]
Penconazole	Oral	LD_{50}	$> 2 \times 10^3$	mg/kg BW	[57]
Prothioconazole	Dermal	LD_{50}	2×10^3	mg/kg BW	[60]
Prothioconazole	Dermal	LD_{50}	$> 2 \times 10^3$	mg/kg BW	[61]
Prothioconazole	Inhalation	LC_{50}	> 5	mg/L	[60]
Prothioconazole	Inhalation	LC_{50}	> 5	mg/L	[61]
Prothioconazole	Oral	LD_{50}	6.2×10^3	mg/kg BW	[51]
Prothioconazole	Oral	LD_{50}	$> 6.2 \times 10^3$	mg/kg BW	[61]
Prothioconazole	Oral	LD_{50}	$> 6.2 \times 10^3$	mg/kg BW	[60]
Tebuconazole	Dermal	LD_{50}	2×10^3	mg/kg BW	[62]
Tebuconazole	Dermal	LD_{50}	$> 20.1 \times 10^2$	mg/kg BW	[63]
Tebuconazole	Inhalation	LC_{50}	> 10	mg/L	[63]
Tebuconazole	Inhalation	LC_{50}	> 2.5	mg/L	[63]
Tebuconazole	Inhalation	LC_{50}	> 5.1	mg/L	[62]
Tebuconazole	Oral	LD_{50}	1.7×10^3	mg/kg BW	[51]
Tebuconazole	Oral	LD_{50}	1.7×10^3	mg/kg BW	[62]
Tebuconazole	Oral	LD_{50}	3.7×10^3	mg/kg BW	[63]
Tetraconazole	Dermal	LD_{50}	2×10^3	mg/kg BW	[64]
Tetraconazole	Dermal	LD_{50}	$> 2 \times 10^3$	mg/kg BW	[65]
Tetraconazole	Inhalation	LC_{50}	> 3.7	mg/L	[65]
Tetraconazole	Inhalation	LC_{50}	> 3.7	mg/L	[64]
Tetraconazole	Oral	LD_{50}	10.3×10^2	mg/kg BW	[66]
Tetraconazole	Oral	LD_{50}	10.3×10^2	mg/kg BW	[65]
Tetraconazole	Oral	LD_{50}	10.3×10^2	mg/kg BW	[64]
Tetraconazole	Oral	LD_{50}	1.25×10^3	mg/kg BW	[67]
Triticonazole	Dermal	LD_{50}	2×10^3	mg/kg BW	[68]
Triticonazole	Dermal	LD_{50}	$> 2 \times 10^3$	mg/kg BW	[69]
Triticonazole	Dermal	LD_{50}	$> 2 \times 10^3$	mg/kg BW	[70]
Triticonazole	Inhalation	LC_{50}	> 3.3	mg/L	[70]
Triticonazole	Inhalation	LC_{50}	> 5.6	mg/L	[69]
Triticonazole	Inhalation	LC_{50}	> 5.6	mg/L	[68]
Triticonazole	Oral	LD_{50}	$> 2 \times 10^3$	mg/kg BW	[69]
Triticonazole	Oral	LD_{50}	$> 2 \times 10^3$	mg/kg BW	[68]
Triticonazole	Oral	LD_{50}	$> 5 \times 10^3$	mg/kg BW	[70]

TP = toxicological parameters; VTP = value of toxicological parameters.; VTP M.U. = measurement unit of value of toxicological parameter; BW = body weight; N/A = data not available.

Table S4. Comparative summary of key outcomes from literature-reported fungicide studies, including triazoles and other fungicides, for contextualization with findings from the current study on selected EU-approved triazole in vitro and *in vivo* toxicity

Source	Type	Analyzed fungicides	Fungicide regulatory context	*In vivo* toxicological parameters	In vitro toxicological parameters	Molecular mechanism of toxicity
[71] *	Review	Bromuconazole<br>Difenoconazole<br>Epoxiconazole<br>Propiconazole<br>Tebuconazole	National (Brazil)	Not accessible	Not applicable (*in vivo* only)	• increased DNA damage;<br>• formation of micronuclei and adducts;<br>• elevated rates of genetic mutations;<br>• induction of oxidative stress.
[72]	Review	Myclobutanil<br>Propiconazole<br>Triadimefon	Not reported	Not applicable (endpoints limited to endocrine activity)	Not applicable (endpoints limited to endocrine activity)	• altered steroidogenesis via inhibition of aromatase.
[73]	Review	Triazole fungicides with available data	Not reported	Not applicable (no toxicity endpoints reported)	Not applicable (no toxicity endpoints reported)	• endocrine disruption;<br>• oxidative stress;<br>• disruption of signaling pathways;<br>• apoptosis.
[74]	Review	Chiral triazole fungicides	Not reported	Not applicable (no toxicity endpoints reported)	Not applicable (no toxicity endpoints reported)	• oxidative stress;<br>• endocrine disruption;<br>• changes in metabolic pathways.
[75] *	Research (HepG2 cells and Sprague–Dawley rats)	Propiconazole	Not reported	Not accessible	Not accessible	• oxidative stress via cytochrome P450;<br>• higher mRNA levels of interleukin-1β, tumor necrosis factor-α, matrix metalloproteinase (MMP)-2, MMP-9, and transforming growth factor-β (TGF-β) than the control.
[76] *	Research (MCF-7 breast cancer cell line)	Other types of fungicides (mancozeb, triphenyltin)	Not reported	Not applicable (in vitro only)	Not accessible	• induced apoptosis;<br>• induced aneuploidy.
[77] *	Research (primary cultured cortical neurons)	Other types of fungicides (ametoctradin, boscalid, cyazofamid, dimethomorph, fenhexamid, kresoxim-methyl, mepanipyrim, metrafenone, pyraclostrobin)	Regional (EU)	Not applicable (in vitro only)	LC_{50} in low micromolar and nanomolar levels	• depolarization of mitochondrial membrane potential
[78] **	Research (male white rats)	Other types of fungicides (phenylpyrrole)	Not reported	Oral LD_{50} > 5000 mg/kg BW	Not applicable (*in vivo* only)	• not analyzed.

* Study not accessible – data was extracted only from abstract; ** Study not in English – data was extracted only from English abstract.

References

1. Rjiba-Touati, K.; Ayed-Boussema, I.; Hamdi, H.; Abid, S. Genotoxic damage and apoptosis in rat glioma (F98) cell line following exposure to bromuconazole. *NeuroToxicology* **2023**, *94*, 108–116, doi:https://doi.org/10.1016/j.neuro.2022.11.006
2. Rjiba-Touati, K.; Ayed-Boussema, I.; Hamdi, H.; Azzebi, A.; Abid, S. Bromuconazole fungicide induces cell cycle arrest and apoptotic cell death in cultured human colon carcinoma cells (HCT116) via oxidative stress process. *Biomarkers* **2022**, *27*, 659–670, doi:https://doi.org/10.1080/1354750X.2022.2098378
3. Kim, M.; Park, W.; Lim, W.; Song, G.; Park, S. Bromuconazole impairs implantation process through cellular stress response in human trophoblast and endometrial cells. *Pestic Biochem Phys* **2025**, *214*, 106632, doi:https://doi.org/10.1016/j.pestbp.2025.106632
4. Rjiba-Touati, K.; Hamdi, H.; M'nassri, A.; Rich, S.; Mokni, M.; Abid, S. Brain injury, genotoxic damage and oxidative stress induced by Bromuconazole in male Wistar rats and in SH-SY5Y cell line. *Biomarkers* **2022**, *27*, 599–607, doi:https://doi.org/10.1080/1354750X.2022.2087002
5. Liu, Y.; Xu, N.; Song, X.; Deng, M.; Sun, R.; Wang, P.; Cao, L. Specific Hepatorenal Toxicity and Cross-Species Susceptibility of Eight Representative Pesticides. *Toxics* **2025**, *13*, 911, doi:https://doi.org/10.3390/toxics13110911
6. Li, R.; Liu, B.; Xu, W.; Yu, L.; Zhang, C.; Cheng, J.; Tao, L.; Li, Z.; Zhang, Y. DNA damage and cell apoptosis induced by fungicide difenoconazole in mouse mononuclear macrophage RAW264.7. *Environ Toxicol* **2022**, *37*, 650–659, doi:https://doi.org/10.1002/tox.23432
7. Wang, T.; Ma, M.; Chen, C.; Yang, X.; Qian, Y. Three widely used pesticides and their mixtures induced cytotoxicity and apoptosis through the ROS-related caspase pathway in HepG2 cells. *Food Chem Toxicol* **2021**, *152*, 112162, doi:https://doi.org/10.1016/j.fct.2021.112162
8. Pan, L.; Lu, L.; Wang, J.; Zheng, C.; Fu, Y.; Xiao, S.; Jin, Y.; Zhuang, S. The fungicide difenoconazole alters mRNA expression levels of human CYP3A4 in HepG2 cells. *Environ Chem Lett* **2017**, *15*, 673–678, doi:https://doi.org/10.1007/s10311-017-0636-0
9. Serra, L.; Estienne, A.; Caria, G.; Ramé, C.; Jolivet, C.; Froger, C.; Henriot, A.; Amalric, L.; Guérif, F.; Froment, P.; et al. In vitro exposure to triazoles used as fungicides impairs human granulosa cells steroidogenesis. *Environ Toxicol Pharm* **2023**, *104*, 104295, doi:https://doi.org/10.1016/j.etap.2023.104295
10. Almedeny, S.A.; Sahib, Z.H.; Al Mukhtar, E.J.; Alkelaby, K.K. Evaluation of the Cytotoxic Effect of the Epoxyconazole and Difenoconazole on Human Colorectal Cancer HCT116 Cell Line. *International Journal of Drug Delivery Technology* **2022**, doi:https://doi.org/10.25258/ijddt.12.2.59
11. Li, Y.-C.; Liu, S.-Y.; Li, H.-R.; Meng, F.-B.; Qiu, J.; Qian, Y.-Z.; Xu, Y.-Y. Use of Transcriptomics to Reveal the Joint Immunotoxicity Mechanism Initiated by Difenoconazole and Chlorothalonil in the Human Jurkat T-Cell Line. *Foods* **2024**, *13*, 34, doi:https://doi.org/10.3390/foods13010034
12. Na, M.-J.; Lee, W.-Y.; Park, H.-J. Difenoconazole Induced Damage of Bovine Mammary Epithelial Cells via ER Stress and Inflammatory Response. *Cells* **2024**, *13*, 1715, doi:https://doi.org/10.3390/cells13201715
13. Wang, X.; Ni, H.; Xu, W.; Wu, B.; Xie, T.; Zhang, C.; Cheng, J.; Li, Z.; Tao, L.; Zhang, Y. Difenoconazole induces oxidative DNA damage and mitochondria mediated apoptosis in SH-SY5Y cells. *Chemosphere* **2021**, *283*, 131160, doi:https://doi.org/10.1016/j.chemosphere.2021.131160
14. Xu, X.-B. Chiral analysis and semi-preparative separation of metconazole stereoisomers by supercritical fluid chromatography and cytotoxicity assessment in vitro. *J Sep Sci* **2024**, *47*, 2300655, doi:https://doi.org/10.1002/jssc.202300655
15. Luo, Y.; Lu, S.; Sun, X.; Gao, Y.; Sun, G.; Yang, M.; Sun, X. Paclobutrazol exposure induces apoptosis and impairs autophagy in hepatocytes via the AMPK/mTOR signaling pathway. *J Biochem Mol Toxic* **2021**, *35*, e22874, doi:https://doi.org/10.1002/jbt.22874
16. Tian, S.; Yan, S.; Meng, Z.; Sun, W.; Yan, J.; Huang, S.; Wang, Y.; Zhou, Z.; Diao, J.; Li, L.; et al. Widening the Lens on Prothioconazole and Its Metabolite Prothioconazole-Desthio: Aryl Hydrocarbon Receptor-Mediated Reproductive Disorders through in Vivo, in Vitro, and in Silico Studies. *Environ Sci Technol* **2022**, *56*, 17890–17901, doi:https://doi.org/10.1021/acs.est.2c06236
17. Hu, L.; Wang, X.; Qian, M.; Zhang, H.; Jin, Y. Impacts of prothioconazole and prothioconazole-desthio on bile acid and glucolipid metabolism: Upregulation of CYP7A1 expression in HepG2 cells. *Pestic Biochem Phys* **2024**, *198*, 105702, doi:https://doi.org/10.1016/j.pestbp.2023.105702
18. Othmène, Y.B.; Kevin, M.; Ahmed, K.; Salem, I.B.; Anissa, B.; Salwa, A.-E.; Christophe, L. Tebuconazole induces ROS-dependent cardiac cell toxicity by activating DNA damage and mitochondrial apoptotic pathway. *Ecotox Environ Safe* **2020**, *204*, 111040, doi:https://doi.org/10.1016/j.ecoenv.2020.111040

19. Kjærstad, M.B.; Taxvig, C.; Nellemann, C.; Vinggaard, A.M.; Andersen, H.R. Endocrine disrupting effects in vitro of conazole antifungals used as pesticides and pharmaceuticals. *Reprod Toxicol* **2010**, *30*, 573–582, doi:https://doi.org/10.1016/j.reprotox.2010.07.009
20. Daniel, S.L.; Hartman, G.L.; Wagner, E.D.; Plewa, M.J. Mammalian Cell Cytotoxicity Analysis of Soybean Rust Fungicides. *B Environ Contam Tox* **2007**, *78*, 474–478, doi:https://doi.org/10.1007/s00128-007-9193-8
21. Othmène, Y.B.; Salem, I.B.; Hamdi, H.; Annabi, E.; Abid-Essefi, S. Tebuconazole induced cytotoxic and genotoxic effects in HCT116 cells through ROS generation. *Pestic Biochem Phys* **2021**, *174*, 104797, doi:https://doi.org/10.1016/j.pestbp.2021.104797
22. Othmène, Y.B.; Kevin, M.; Anissa, B.; Ahmed, K.; Intidhar, B.S.; Manel, B.; Salwa, A.-E.; Christophe, L. Triazole fungicide tebuconazole induces apoptosis through ROS-mediated endoplasmic reticulum stress pathway. *Environ Toxicol Pharm* **2022**, *94*, 103919, doi:https://doi.org/10.1016/j.etap.2022.103919
23. Nie, J.; Zhou, J.; Shen, Y.; Lin, R.; Hu, H.; Zeng, K.; Bi, H.; Huang, M.; Yu, L.; Zeng, S.; et al. Studies on the interaction of five triazole fungicides with human renal transporters in cells. *Toxicol in vitro* **2023**, *88*, 105555, doi:https://doi.org/10.1016/j.tiv.2023.105555
24. Bülbül, E.; Özhan, G.; Bülbül, E.; Bülbül, G.Ö.E.; Özhan, G. Cytotoxic effects of triazole fungucides. *J Fac Pharm Istanbul Univ* **2012**, *1*, 23–31, https://dergipark.org.tr/en/download/article-file/5149.
25. Andrioli, N.B.; Nieves, M.; Poltronieri, M.; Bonzon, C.; Chaufan, G. Genotoxic effects induced for sub-cytotoxic concentrations of tebuconazole fungicide in HEp-2 cell line. *Chem-Biol Interact* **2023**, *373*, 110385, doi:https://doi.org/10.1016/j.cbi.2023.110385
26. Barrón Cuenca, J.; de Oliveira Galvão, M.F.; Ünlü Endirlik, B.; Tirado, N.; Dreij, K. In vitro cytotoxicity and genotoxicity of single and combined pesticides used by Bolivian farmers. *Environ Mol Mutagen* **2022**, *63*, 4–17, doi:https://doi.org/10.1002/em.22468
27. Leite, F.G.; Silva, C.d.P.M.; Miranda, R.G.; Dorta, D.J. Comparison of in vitro toxicity in HepG2 cells: Toxicological role of Tebuconazole-tert-butyl-hydroxy in exposure to the fungicide Tebuconazole. *Pestic Biochem Phys* **2024**, *202*, 105954, doi:https://doi.org/10.1016/j.pestbp.2024.105954
28. Kwon, H.-C.; Kim, D.-H.; Jeong, C.-H.; Kim, Y.-J.; Han, J.-H.; Lim, S.-J.; Shin, D.-M.; Kim, D.-W.; Han, S.-G. Tebuconazole Fungicide Induces Lipid Accumulation and Oxidative Stress in HepG2 Cells. *Foods* **2021**, *10*, 2242, doi:https://doi.org/10.3390/foods10102242
29. Serra, L.; Estienne, A.; Bongrani, A.; Ramé, C.; Caria, G.; Froger, C.; Jolivet, C.; Henriot, A.; Amalric, L.; Corbin, E.; et al. The epoxiconazole and tebuconazole fungicides impair granulosa cells functions partly through the aryl hydrocarbon receptor (AHR) signalling with contrasted effects in obese, normo-weight and polycystic ovarian syndrome (PCOS) patients. *Toxicol Rep* **2024**, *12*, 65–81, doi:https://doi.org/10.1016/j.toxrep.2023.12.009
30. Zhou, J.; Zhang, J.; Li, F.; Liu, J. Triazole fungicide tebuconazole disrupts human placental trophoblast cell functions. *J Hazard Mater* **2016**, *308*, 294–302, doi:https://doi.org/10.1016/j.jhazmat.2016.01.055
31. Sevim, Ç.; Taghizadehghalehjoughi, A.; Kara, M. Effects of chlorpyrifos-methyl, chlormequat, deltamethrin, glyphosate, pirimiphos-methyl, tebuconazole and their mixture on oxidative stress and toxicity in HUVEC cell line. *Istanbul J Pharm* **2021**, *51*, 183–190, doi:https://doi.org/10.26650/IstanbulJPharm.2021.881724
32. Xu, J.; Xiong, H.; Zhang, X.; Muhayimana, S.; Liu, X.; Xue, Y.; Huang, Q. Comparative cytotoxic effects of five commonly used triazole alcohol fungicides on human cells of different tissue types. *J Environ Sci Heal B* **2020**, *55*, 438–446, doi:https://doi.org/10.1080/03601234.2019.1709377
33. Lee, W.-Y.; Lee, R.; Park, H.-J. Tebuconazole Induces ER-Stress-Mediated Cell Death in Bovine Mammary Epithelial Cell Lines. *Toxics* **2023**, *11*, 397, doi:https://doi.org/10.3390/toxics11040397
34. Sanchez, C.L.; Souders, C.L.; Pena-Delgado, C.J.; Nguyen, K.T.; Kroyter, N.; Ahmadie, N.E.; Aristizabal-Henao, J.J.; Bowden, J.A.; Martyniuk, C.J. Neurotoxicity assessment of triazole fungicides on mitochondrial oxidative respiration and lipids in differentiated human SH-SY5Y neuroblastoma cells. *NeuroToxicology* **2020**, *80*, 76–86, doi:https://doi.org/10.1016/j.neuro.2020.06.009
35. Jeong, S.A.; Song, J.; Ham, J.; An, G.; Song, G.; Lim, W. Tetraconazole interrupts mitochondrial function and intracellular calcium levels leading to apoptosis of bovine mammary epithelial cells. *Pestic Biochem Phys* **2023**, *191*, 105366, doi:https://doi.org/10.1016/j.pestbp.2023.105366
36. de Jong, E.; Barenys, M.; Hermsen, S.A.B.; Verhoef, A.; Ossendorp, B.C.; Bessems, J.G.M.; Piersma, A.H. Comparison of the mouse Embryonic Stem cell Test, the rat Whole Embryo Culture and the Zebrafish Embryotoxicity Test as alternative methods for developmental toxicity testing of six 1,2,4-triazoles. *Toxicol Appl Pharm* **2011**, *253*, 103–111, doi:https://doi.org/10.1016/j.taap.2011.03.014

37. Pesticide Properties DataBase. Bromuconazole. Available online: https://sitem.herts.ac.uk/aeru/ppdb/en/Reports/97.htm#3 (accessed on 30 October 2025).
38. European Food Safety Authority. Conclusion on the peer review of the pesticide risk assessment of the active substance bromuconazole. *EFSA Journal* **2010**, *8*, 1704, doi:https://doi.org/10.2903/j.efsa.2010.1704
39. European Food Safety Authority. Conclusion regarding the peer review of the pesticide risk assessment of the active substance bromuconazole. *EFSA Journal* **2008**, *6*, 136r, doi:https://doi.org/10.2903/j.efsa.2010.1704
40. Rjiba-Touati, K.; Hamdi, H.; M'nassri, A.; Guedri, Y.; Mokni, M.; Abid, S. Bromuconazole caused genotoxicity and hepatic and renal damage via oxidative stress process in Wistar rats. *Environ Sci Pollut R* **2022**, *29*, 14111–14120, doi:https://doi.org/10.1007/s11356-021-16091-8
41. Hariyadi, H.R. The presence of bromuconazole fungicide pollutant in organic waste anaerobic fermentation. *IOP Conference Series: Earth and Environmental Science* **2017**, *60*, 012022, doi:https://doi.org/10.1088/1755-1315/60/1/012022
42. Osman, A.H.; Elshama, S.S.; Osman, A.S.; El-Hameed, A.K.A. Toxicological and pathological evaluation of prolonged bromuconazole fungicide exposure in male rats. *Med J Cairo Univ* **2011**, *79*, 555–564, https://scholar.cu.edu.eg/?q=yomnakhaled/files/cairo_univeristy_journal.pdf.
43. Pesticide Properties DataBase. Difenoconazole. Available online: https://sitem.herts.ac.uk/aeru/ppdb/en/Reports/230.htm#3 (accessed on 30 October 2025).
44. European Food Safety Authority. Conclusion on the peer review of the pesticide risk assessment of the active substance difenoconazole. *EFSA Journal* **2011**, *9*, 1967, doi:https://doi.org/10.2903/j.efsa.2011.1967
45. Mohamed, R.E.s.; El-Sayed, M.M.; Arief, M.M.; Mahmoud, A.A. Evaluation of the ameliorative effect of Cinnamon cassia against metabolic disorder and thyroid hormonal disruption following treatment with difenoconazole fungicide in the male albino rats. *Egypt J Chem* **2022**, *65*, 55–67, doi:https://doi.org/10.21608/ejchem.2021.76914.3766
46. Khwanes, S.A.; Mohamed, R.A.; Ibrahim, K.A.; Abd El-Rahman, H.A. Ginger reserves testicular spermatogenesis and steroidogenesis in difenoconazole-intoxicated rats by conducting oxidative stress, apoptosis and proliferation. *Andrologia* **2022**, *54*, e14241, doi:https://doi.org/10.1111/and.14241
47. Pesticide Properties DataBase. Mefentrifluconazole. Available online: https://sitem.herts.ac.uk/aeru/ppdb/en/Reports/3098.htm#3 (accessed on 30 October 2025).
48. Tesh, S.A.; Tesh, J.M.; Fegert, I.; Buesen, R.; Schneider, S.; Mentzel, T.; van Ravenzwaay, B.; Stinchcombe, S. Innovative selection approach for a new antifungal agent mefentrifluconazole (Revysol®) and the impact upon its toxicity profile. *Regul Toxicol Pharm* **2019**, *106*, 152–168, doi:https://doi.org/10.1016/j.yrtph.2019.04.009
49. Pesticide Properties DataBase. Metconazole. Available online: https://sitem.herts.ac.uk/aeru/ppdb/en/Reports/451.htm#3 (accessed on 30 October 2025).
50. United States Environmental Protection Agency. Pesticide Fact Sheet - Metconazole. **2007**, https://www3.epa.gov/pesticides/chem_search/reg_actions/registration/fs_PC-125619_01-Sep-07.pdf.
51. Klix, M.B.; Verreet, J.-A.; Beyer, M. Comparison of the declining triazole sensitivity of Gibberella zeae and increased sensitivity achieved by advances in triazole fungicide development. *Crop Prot* **2007**, *26*, 683–690, doi:https://doi.org/10.1016/j.cropro.2006.06.006
52. European Food Safety Authority. Conclusion on the peer review of the pesticide risk assessment of the active substance paclobutrazol. *EFSA Journal* **2010**, *8*, 1876, doi:https://doi.org/10.2903/j.efsa.2010.1876
53. Pesticide Properties DataBase. Paclobutrazol. Available online: https://sitem.herts.ac.uk/aeru/ppdb/en/Reports/504.htm#3 (accessed on 30 October 2025).
54. Xu, M.; Yang, F. Integrated gender-related effects of profenofos and paclobutrazol on neurotransmitters in mouse. *Ecotox Environ Safe* **2020**, *190*, 110085, doi:https://doi.org/10.1016/j.ecoenv.2019.110085
55. Li, X.; Lian, T.; Su, B.; Liu, H.; Wang, Y.; Wu, X.; He, J.; Wang, Y.; Xu, Y.; Yang, S.; et al. Construction of a physiologically based pharmacokinetic model of paclobutrazol and exposure estimation in the human body. *Toxicology* **2024**, *505*, 153841, doi:https://doi.org/10.1016/j.tox.2024.153841
56. Yue, K.; Liu, Z.; Pi, Z.; Li, H.; Wang, Y.; Song, F.; Liu, Z. Network Pharmacology Combined with Metabolomics Approach to Investigate the Toxicity Mechanism of Paclobutrazol. *Chem Res Toxicol* **2022**, *35*, 626–635, doi:https://doi.org/10.1021/acs.chemrestox.1c00404
57. Pesticide Properties DataBase. Penconazole. Available online: https://sitem.herts.ac.uk/aeru/ppdb/en/Reports/509.htm#3 (accessed on 30 October 2025).
58. Food and Agriculture Organization of the United Nations. *Toxicological Evaluation of Penconazole*; 2015.

59. El-Shershaby, A.E.-F.M.; Lashein, F.E.-D.M.; Seleem, A.A.; Ahmed, A.A. Toxicological potential of penconazole on early embryogenesis of white mice Mus musculus in either pre- or post-implantation exposure. *Environ Sci Pollut R* **2020**, *27*, 9943–9956, doi:https://doi.org/10.1007/s11356-020-07637-3
60. Pesticide Properties DataBase. Prothioconazole. Available online: https://sitem.herts.ac.uk/aeru/ppdb/en/Reports/559.htm#3 (accessed on 30 October 2025).
61. Australian Pesticides and Veterinary Medicines Authority. Evaluation of the new active prothioconazole in the product Redigo fungicidal seed treatment. **2007**, https://www.apvma.gov.au/sites/default/files/publication/13941-prs-prothioconazole.pdf.
62. Pesticide Properties DataBase. Tebuconazole. Available online: https://sitem.herts.ac.uk/aeru/ppdb/en/Reports/610.htm#3 (accessed on 30 October 2025).
63. Solera ATO, L. *Safety Data Sheet - Tebuconazole*; 2015.
64. Pesticide Properties DataBase. Tetraconazole. Available online: https://sitem.herts.ac.uk/aeru/ppdb/en/Reports/626.htm#3 (accessed on 30 October 2025).
65. European Food Safety Authority. Conclusion regarding the peer review of the pesticide risk assessment of the active substance tetraconazole. *EFSA Journal* **2008**, *152*, 1–86, doi:https://doi.org/10.2903/j.efsa.2008.152r
66. United States Environmental Protection Agency. Pesticide Fact Sheet - Tetraconazole. **2005**, https://www3.epa.gov/pesticides/chem_search/reg_actions/registration/fs_PC-120603_01-Apr-05.pdf.
67. Abbassy, M.A.; Marzouk, M.A.; Nasr, H.M.; Mansy, A. Effect of imidacloprid and tetraconazole on various hematological and biochemical parameters in male albino rats (Rattus norvegious). *J Pol Sci Pub Aff* **2014**, *2*, 2332–0761.1000122, doi:https://doi.org/10.4172/2332-0761.1000122
68. Pesticide Properties DataBase. Triticonazole. Available online: https://sitem.herts.ac.uk/aeru/ppdb/en/Reports/673.htm#3 (accessed on 30 October 2025).
69. European Food Safety Authority. Conclusion regarding the peer review of the pesticide risk assessment of the active substance triticonazole. *EFSA Journal* **2005**, *3*, 33ar, doi:https://doi.org/10.2903/j.efsa.2005.33ar
70. United States Environmental Protection Agency. Pesticide Fact Sheet - Triticonazole. **2005**, https://www3.epa.gov/pesticides/chem_search/cleared_reviews/csr_PC-125620_1-Nov-00_a.pdf.
71. Correia, L.V.B.; de Aguiar, G.C.; Pereira, A.M.R.d.S.; Thomaz, L.d.S.C.; de Oliveira, I.C.C.d.S.; Mari, R.d.B.; Perobelli, J.E.; Ribeiro, D.A.; da Silva, R.C.B. Triazole fungicides induce genotoxicity via oxidative stress in mammals in vivo: a comprehensive review. *Reviews on Environmental Health* **2025**, doi:https://doi.org/10.1515/reveh-2025-0051
72. Paul Friedman, K.; Papineni, S.; Marty, M.S.; Yi, K.D.; Goetz, A.K.; Rasoulpour, R.J.; Kwiatkowski, P.; Wolf, D.C.; Blacker, A.M.; Peffer, R.C. A predictive data-driven framework for endocrine prioritization: a triazole fungicide case study. *Critical Reviews in Toxicology* **2016**, *46*, 785–833, doi:https://doi.org/10.1080/10408444.2016.1193722
73. Sharma, S.; Pandey, G. Understanding the impact of triazoles on female fertility and embryo development: Mechanisms and implications. *Toxicol Rep* **2025**, *14*, 101948, doi:https://doi.org/10.1016/j.toxrep.2025.101948
74. Wang, N.; Liu, Y.; Li, J.; Xu, L.; Liang, X. Advances in Selective Bioactivity and Toxicity of Chiral Triazole Fungicides and Their Selective Behavior in Mammals. *Chirality* **2025**, *37*, e70055, doi:https://doi.org/10.1002/chir.70055
75. Kwon, H.C.; Sohn, H.; Kim, D.H.; Shin, D.M.; Jeong, C.H.; Chang, Y.H.; Yune, J.H.; Kim, Y.J.; Kim, D.-W.; Kim, S.H.; et al. In Vitro and In Vivo Study on the Toxic Effects of Propiconazole Fungicide in the Pathogenesis of Liver Fibrosis. *J Agr Food Chem* **2021**, *69*, 7399–7408, doi:https://doi.org/10.1021/acs.jafc.1c01086
76. N. Lin, V.F.G. In vitro studies of cellular and molecular developmental toxicity of adjuvants, herbicides, and fungicides commonly used in Red River Valley, M innesota. *Journal of Toxicology and Environmental Health, Part A* **2000**, *60*, 423–439, doi:https://doi.org/10.1080/00984100050033494
77. Regueiro, J.; Olguín, N.; Simal-Gándara, J.; Suñol, C. Toxicity evaluation of new agricultural fungicides in primary cultured cortical neurons. *Environmental Research* **2015**, *140*, 37–44, doi:https://doi.org/10.1016/j.envres.2015.03.013
78. Safandeev, V.; Beloedova, N.; Poroshin, M.; Bogdanova, A.; Sinitskaya, T. Characterization of a phenylpyrrole derivative fungicide in an acute oral toxicology study in rats. *Health care of Kyrgyzs tan* **2023**, 54–58, doi:https://dx.doi.org/10.51350/zdravkg2023.1.2.7.54.58
